# A pipeline for structure determination of *in vivo*-grown crystals using *in cellulo* diffraction

**DOI:** 10.1107/S2059798316002369

**Published:** 2016-03-30

**Authors:** Marion Boudes, Damià Garriga, Andrew Fryga, Tom Caradoc-Davies, Fasséli Coulibaly

**Affiliations:** a Infection and Immunity Program, Monash Biomedicine Discovery Institute and Department of Biochemistry and Molecular Biology, Monash University, Melbourne, VIC 3800, Australia; b Faculty of Medicine, Nursing and Health Sciences, FlowCore, Monash University, Melbourne, VIC 3800, Australia; c The Australian Synchrotron, Clayton, Melbourne, VIC 3800, Australia

**Keywords:** microcrystallography, *in cellulo *diffraction, *in vivo* crystallization, polyhedrin

## Abstract

While structure determination from micrometre-sized crystals used to represent a challenge, serial X-ray crystallography on microfocus beamlines at synchrotron and free-electron laser facilities greatly facilitates this process today for microcrystals and nanocrystals. In addition to typical microcrystals of purified recombinant protein, these advances have enabled the analysis of microcrystals produced inside living cells. Here, a pipeline where crystals are grown in insect cells, sorted by flow cytometry and directly analysed by X-ray diffraction is presented and applied to *in vivo*-grown crystals of the recombinant CPV1 polyhedrin. When compared with the analysis of purified crystals, *in cellulo* diffraction produces data of better quality and a gain of ∼0.35 Å in resolution for comparable beamtime usage. Importantly, crystals within cells are readily derivatized with gold and iodine compounds through the cellular membrane. Using the multiple isomorphous replacement method, a near-complete model was autobuilt from 2.7 Å resolution data. Thus, in favourable cases, an *in cellulo* pipeline can replace the complete workflow of structure determination without compromising the quality of the resulting model. In addition to its efficiency, this approach maintains the protein in a cellular context throughout the analysis, which reduces the risk of disrupting transient or labile interactions in protein–protein or protein–ligand complexes.

## Abstract 2

A streamlined pipeline for structure determination is described that applies *in cellulo* X-ray diffraction analysis to crystal-containing cells isolated by flow cytometry. Application of this pipeline to *in vivo*-grown crystals of the recombinant CPV1 polyhedrin shows that *in cellulo* analysis is more efficient, is compatible with experimental phasing and does not compromise the quality of the final model.

## 1. Introduction   

Large structural genomics consortia and platforms have pushed the development of high-throughput pipelines for the structure determination of biological macromolecules. Miniaturization and automation of crystallogenesis has now been adopted by most medium-to-large research centres, greatly accelerating the typical structural biology workflow (Abola *et al.*, 2000 ▸). Despite these advances, data from structural biology consortia show that the production of diffraction-quality crystals remains the main bottleneck in X-ray crystallography. For instance, the success rate in generating crystals suitable for an X-ray diffraction experiment is estimated at only 27% by the Protein Structure Initiative (data from the TargetTrack database; Chen *et al.*, 2004 ▸; http://sbkb.org/tt/).

While research to understand and facilitate protein crystallization has primarily focused on *in vitro* crystallization of model proteins and colloidal material (Durbin & Feher, 1986 ▸; Stradner *et al.*, 2004 ▸), complementary insights have recently been obtained from structural analysis of *in vivo*-grown crystals (Chiu *et al.*, 2012 ▸). These studies revealed the architecture of some of these *in vivo*-grown crystals and provided a molecular framework to investigate how these proteins spontaneously crystallize in the complex environment of the cells in which they are expressed (Chiu *et al.*, 2012 ▸).

Such naturally occurring crystals are relatively common and are found in organisms spanning all kingdoms of life (Doye & Poon, 2006 ▸). They often represent a form of storage for abundant and toxic proteins. Well characterized examples include the virulence factors of insect pathogens such as poxviruses (Bergoin *et al.*, 1976 ▸; Chiu *et al.*, 2015 ▸) and *Bacillus thuringiensis* (Schnepf *et al.*, 1998 ▸), structural proteins such as trichocysts of *Paramecium* (Sperling *et al.*, 1987 ▸) and Woronin bodies in filamentous fungi (Yuan *et al.*, 2003 ▸), and the storage of active proteins such as alcohol oxidase in yeast peroxisomes (Veenhuis *et al.*, 2003 ▸), protein vesicles in plant seeds (Lott & Spitzer, 1980 ▸) and insulin-secretory granules in mammals (Dodson & Steiner, 1998 ▸).

*In vivo* crystallization has also been reported for the recombinant expression of heterologous polyhedrin proteins in insect cells, resulting in the formation of robust intracellular crystals of the cypovirus polyhedrin proteins that recapitulate their role in the infectious cycle as a crystalline armour around infectious particles (Mori *et al.*, 1993 ▸). Perhaps more unexpectedly, a number of proteins that do not form crystals in their functional context also produce *in vivo* crystals (Fan *et al.*, 1996 ▸; Koopmann *et al.*, 2012 ▸; Redecke *et al.*, 2013 ▸; Schönherr *et al.*, 2015 ▸; Tsutsui *et al.*, 2015 ▸). Thus, a wide range of proteins with unrelated functions, structures and physicochemical properties have now been reported to crystallize *in vivo*, suggesting that this strategy could be routinely used for structure determination alongside classical crystallogenesis approaches (Duszenko *et al.*, 2015 ▸).

The use of *in vivo*-grown crystals in structural biology has long been limited by their very small size owing to the restricted amount of intracellular protein. The advent of modern microcrystallography approaches has alleviated this limitation (Evans *et al.*, 2011 ▸; Smith *et al.*, 2012 ▸; Boudes *et al.*, 2014 ▸), and microfocus beamlines are now available at most third-generation synchrotron sources (Boudes *et al.*, 2014 ▸). More recently, serial crystallography has provided new methodologies to allow the collection of complete data sets from hundreds of microcrystals using X-ray radiation produced by synchrotron facilities (Gati *et al.*, 2014 ▸) and free-electron lasers (XFELs; Redecke *et al.*, 2013 ▸).

However, analysis of *in vivo*-grown microcrystals remains time-intensive owing to inefficient purification from the cell, degradation upon cell lysis and complications in the diffraction experiments, including finding and centring crystals. To overcome some of these difficulties, *in cellulo* data collection, where whole crystal-containing cells are exposed to the X-ray beam, has been used to analyse CPV18 polyhedrin crystals in insect cells and the coral protein Xpa in mammalian cells using X-ray crystallography (Axford *et al.*, 2014 ▸; Tsutsui *et al.*, 2015 ▸) and the Cry3A toxin in *B. thuringiensis* cells using an XFEL (Sawaya *et al.*, 2014 ▸).

Difficulties in handling and phasing remain the major barriers to the wider adoption of microcrystallography. Here, we present a simple pipeline for structure determination using *in vivo*-grown crystals. The pipline is based on *in cellulo* diffraction analysis from crystal-containing cells sorted by flow cytometry. We show that this workflow is more efficient than data collection from purified crystals and may generate data of better quality. The protocol is compatible with experimental phasing by the multiple isomorphous replacement (MIR) method, simplifying the path from expression to structure determination. Using *in vivo* crystals of the *Bombyx mori* CPV1 (CPV1) polyhedrin as a model system, we show that *de novo* structure determination can be carried out at a resolution of 1.5 Å in ∼8 days from expression to refinement using data measured on a standard microfocus beamline.

## 2. Pipeline overview   

**Figure 1:**
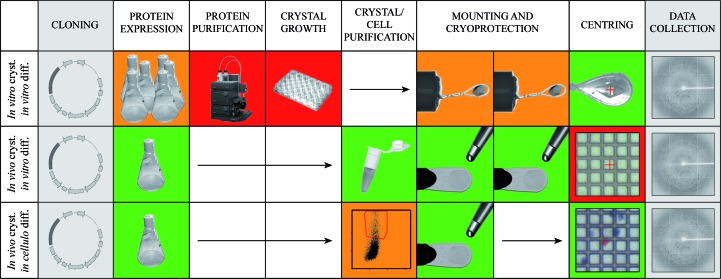
Overview of different structure-determination pipelines. Comparison of classical crystallography methods with the *in cellulo* pipeline. An arbitrary colour scale ranging from green (least demanding) to red (most demanding) indicates the relative level of skills and/or resources required for each step between the three workflows. Steps that are identical in all workflows are shaded in grey. For the *in vitro* crystallization pipeline (top row), the protein is expressed in large amounts, purified and used to find and optimize crystallization conditions. Crystals are then cryoprotected, harvested with nylon loops and flash-cooled by immersion in liquid nitrogen. In the *in vivo* crystallization approach (middle row), crystals are grown in the cells that express the protein, bypassing protein purification and the crystallization steps. In the classical microcrystallography approach, cells are lysed and crystals are purified by centrifugation. After mounting on a support, typically a grid mesh, crystals are further incubated with an appropriate cryoprotectant solution, the excess liquid is removed and the support is then flash-cooled. Once the support has been loaded onto a goniometer, each crystal has to be accurately centred in the X-ray beam to ensure that it is properly exposed. In an *in cellulo* pipeline (bottom row), crystals are produced as in the *in vivo* approach, but the host cells are not lysed. Instead, crystal-containing cells are sorted by flow cytometry, stained with trypan blue and mounted on a support. No cryoprotectant solution is required. Cell sorting is indicated in orange since it is straightforward and inexpensive if available through a shared instrument/facility, but otherwise requires additional resources and training. Once at the beamline, the stain and the larger size of the cells compared with crystals facilitate the identification of crystal-containing cells and alignment with the beam. The image of the automated liquid-chromatography system is courtesy of GE Healthcare AB, Uppsala, Sweden.

The proposed pipeline has been designed with a particular focus on removing or simplifying steps in the structure-determination workflow that require specialized knowhow such as crystal handling and sample preparation. A schematic representation of the pipeline is displayed in Fig. 1 ▸. A suspension of cultured Sf9 cells overexpressing a recombinant protein is screened for the formation of *in vivo* crystals, typically by bright-field microscopy. In most cases, cells containing microcrystals are mixed with ‘empty’ cells because of variability in the kinetics and the level of protein expression. To overcome this heterogeneity, which decreases the efficiency of beamtime use, we have introduced a step of cell sorting by flow cytometry to isolate the crystal-containing population. Flow cytometry is a laser-based technology for counting and sorting cells. While it is not routinely used in structural biology, this tool is widely available through shared instruments or technology platforms in research institutions because of its extensive use in disciplines such as immunology, developmental biology and cancer research. If such a shared instrument is not available, the sorting step uses the simplest sorting parameter (*i.e.* not fluorescence-assisted), which means that it does not require advanced skills beyond instrument training and is compatible with the new generation of automated benchtop cell sorters. After sorting, cells are stained to facilitate their visualization throughout subsequent steps and particularly for handling at the beamline, where cameras have poorer resolution compared with laboratory microscopes. Cells are then directly pipetted onto a mesh grid support and flash-cooled in liquid nitrogen. In the case of the CPV1 polyhedra studied here, cryoprotection is not required prior to cooling. Finally, the mesh is mounted on a standard gonio­meter at a synchrotron microfocus beamline. Isolated cells are sequentially centred in the X-ray beam to collect diffraction data *in cellulo*. For *de novo* structure determination using experimental phasing, the cells are incubated in a saturated solution of the heavy atom of choice prior to mounting on the micromesh support and flash-cooling.

## 3. Cell sorting   

**Figure 2:**
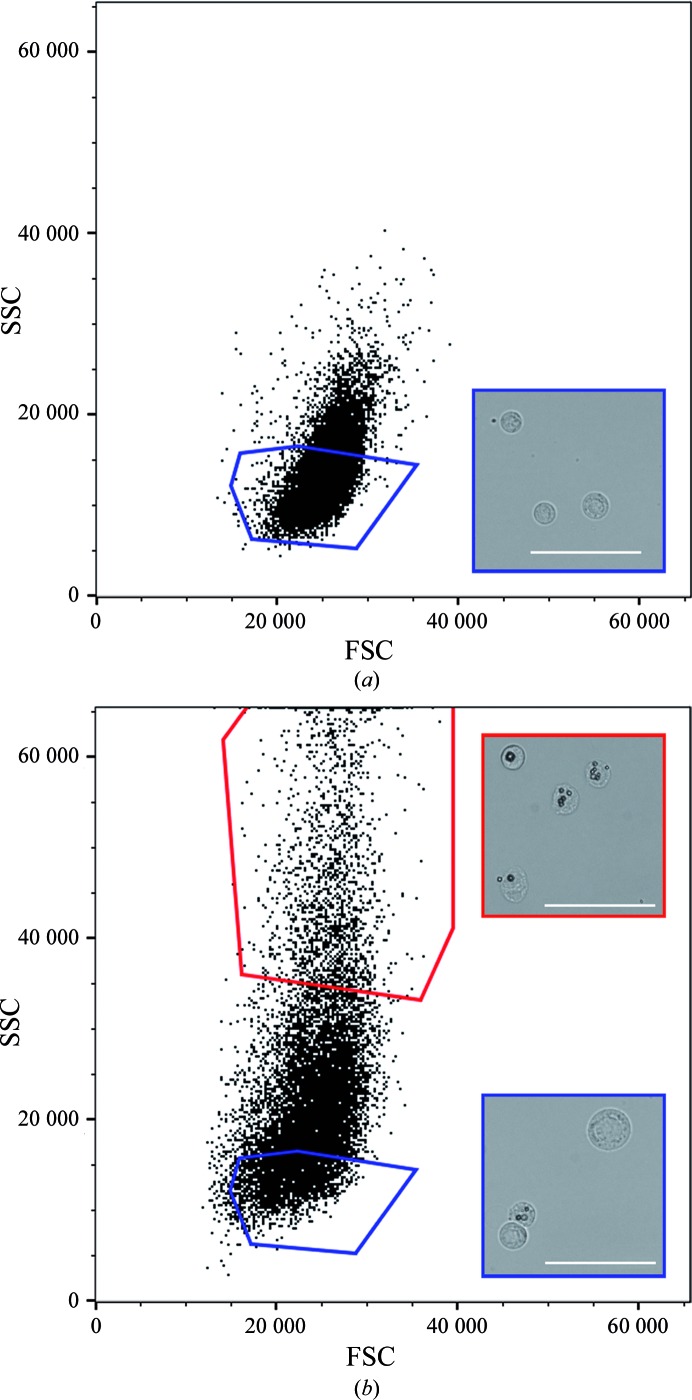
Cell sorting. Representative flow-cytometry scatter plots from (*a*) a non-infected cell sample (mock), which does not contain any crystals, and (*b*) Sf9 cells infected by a recombinant baculovirus coding for the CPV1 polyhedrin. Differences in the scattering pattern between the two populations allowed gating of the crystal-containing cell population (red gate, high SSC values). For comparison, a second population at lower values of SSC was also gated and collected. Representative optical microscopy images of each population are shown in the insets next to the corresponding gate. Scale bars, 50 µm. SSC, side light scattering; FSC, forward light scattering.

Inspection of harvested Sf9 cells by bright-field microscopy reveals that an average of 65% of the cells contained visible crystals. When these cells are analysed and sorted by flow cytometry using correlated measurement of light forward-scattering and side-scattering, a distinct population of cells can be identified with high values of side-scattering, which is indicative of an increased internal complexity of the cells. The protein in the crystals is assumed to be the target since the crystal-containing population is only seen in cells infected by the recombinant baculovirus and is absent in cells infected by a control baculovirus (data not shown). If not known before, the identity of the protein in the crystal can also be confirmed at this stage by isolation of the crystals for mass spectrometry or Western blot analysis. Bright-field microscopy confirms that every cell in this population contains polyhedrin crystals (Fig. 2 ▸). In contrast, populations at low side-scattering values include only ∼30% of crystal-containing cells.

During sorting, optimization of the gates ensures that the selected population consists exclusively of crystal-containing cells. Although this gating excludes the majority of crystal-containing cells, enough cells are sorted from 10 ml of culture to prepare >1000 meshes in a typical 1 h run.

Owing to the transfer from cell-culture medium into PBS and variations in temperature, the viability of the Sf9 cells is lost during the cell-sorting step. Nevertheless, imaging of sorted samples suggests that the physical integrity of the cells is maintained. In fact, lysed cells and cell debris from the culture are efficiently removed by gating for the high side-scattering population, contributing to the quality of the final sample.

## 4. Sample preparation, mounting and handling on the beamline   

To enhance the visualization of cells in the sample, the sorted cells were concentrated by centrifugation to approximately 10^7^ cells ml^−1^ and mixed with an equal volume of trypan blue stain. This dye was selected because of its ubiquitous use in cell culture to assess viability. In our experimental conditions, the dye was readily incorporated into most Sf9 cells owing to a loss of viability after sorting. To mount the sample for diffraction experiments, a volume of 0.5 µl of sorted cells was pipetted onto a MiTeGen micromesh used as a static stand. Excess liquid was removed by blotting with a paper wick to facilitate centring and to reduce background scattering. Reducing the thickness of the solvent around cells is critical to reduce parallax effects that complicate the process of alignment between the crystal, the beam path and the goniometer rotation axis. A thin film also minimizes the background caused by scattering of X-rays by liquid in the beam path.

**Figure 3:**
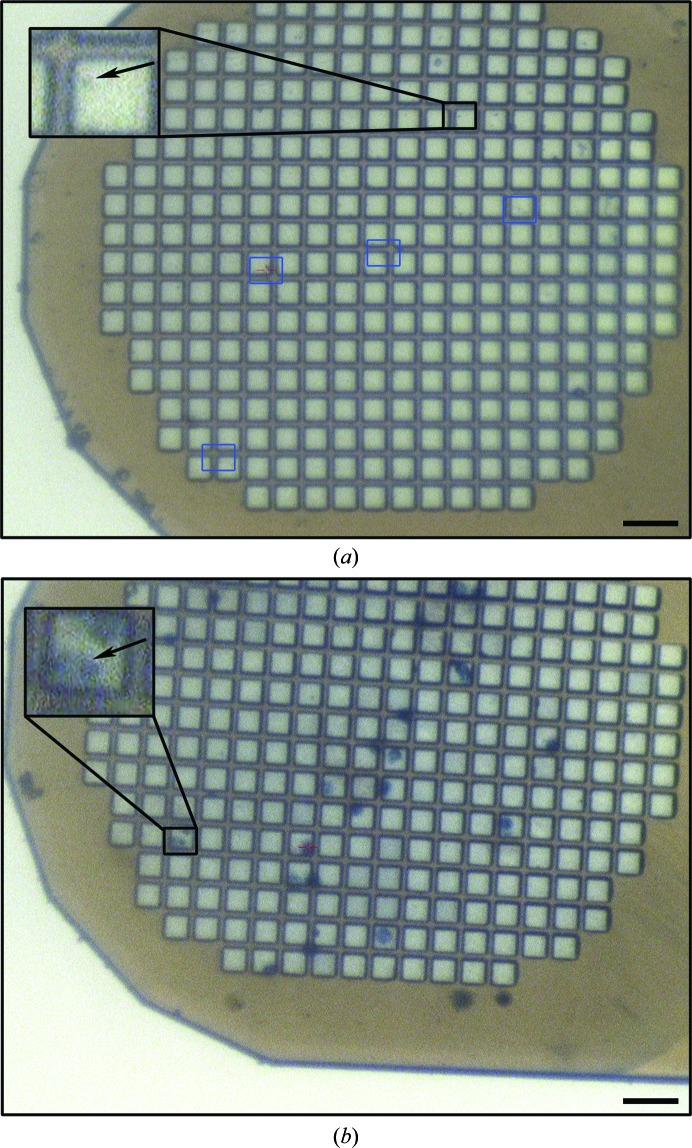
Comparison of trypan-stained cells and purified crystals loaded onto a micromesh support. Images are shown of (*a*) a mesh loaded with purified polyhedra and (*b*) a mesh loaded with trypan-stained polyhedra-containing cells, as displayed in the crystal-centring interface at the Australian Synchrotron MX2 beamline. The scale bar corresponds to 50 µm. Insets are close-ups of the areas indicated by the black boxes, with an arrow pointing to the position of the crystal. In (*a*), the blue and black boxes indicate examples of positions with diffracting polyhedrin crystals. Note that the polyhedrin microcrystals are hardly visible on the mesh in both panels.

**Figure 4:**
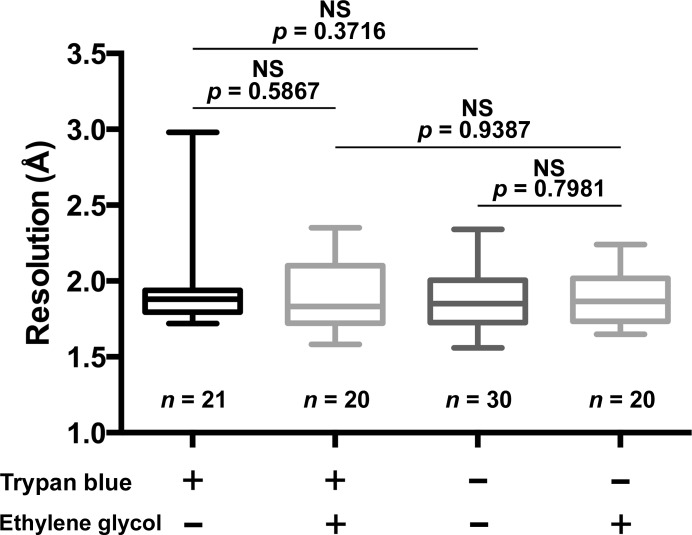
Effect of cryoprotectant and trypan blue staining on *in cellulo* crystal diffraction. Distribution of the maximum resolutions achieved from individual crystal-containing cells with or without trypan blue and ethylene glycol. The resolution limit is estimated using *DISTL* as described in §[Sec sec8]8 and is represented as column graphs. For each condition, boxes represent the 25th and 75th percentiles, the central line is the average value and whiskers indicate the minimum and maximum values. The *p* values are displayed and indicate no significant differences between the four conditions.

Cells treated with trypan blue were readily visible on the mesh both on an inverted microscope and at the beamline fitted with an on-axis microscope (Navitar; Fig. 3 ▸ *b*). Thus, staining greatly improves the ability to monitor the distribution of cells on micromeshes and their alignment with the microfocus beam. This latter step is typically the most time-consuming process in data collection for crystals that are approximately the size of the beam. To quantify this improvement in data-collection efficiency, we define the hit rate as the proportion of successful diffraction tests, irrespective of the diffraction limit. *In cellulo* and purified crystals had hit rates of 95.9% (two runs; *n* = 21 and *n* = 77) and 76.4% (two runs; *n* = 30 and *n* = 93), respectively. The centring of stained cells was more accurate than that of purified crystals, with 26% more diffracting crystals successfully aligned when analysing crystal-containing cells. These data also confirm that staining of the cells does not affect the quality or the resolution limit of the crystal diffraction (Fig. 4 ▸). The hit rates and the proportion of multiple crystals in the beam depend on the relative sizes of the crystals and the beam. Here, the crystals are cubes with edges of ∼5–15 µm and the beam is collimated to a cross-section of 10 × 10 µm. A larger beam will increase the hit rate but also the probability of hitting multiple crystals. For a given beam size, smaller crystals will simultaneously decrease the hit rate and increase the risk of hitting multiple crystals if the crystal density is high.

To further streamline this workflow, we investigated whether cryoprotection is required when crystals are embedded in the cell. In the case of purified crystals of the CPV1 polyhedrin, the addition of ethylene glycol was needed to collect consistent, high-resolution diffraction data. In contrast, omission of the cryoprotectant additive had no significant effect on *in cellulo* crystals. Samples from cells treated with and without incubation in 50% ethylene glycol diffracted to similar resolution limits (Fig. 4 ▸), which were comparable to the resolutions reported for purified and cryoprotected crystals (Coulibaly *et al.*, 2007 ▸).

## 5. *In cellulo* diffraction   

**Figure 5:**
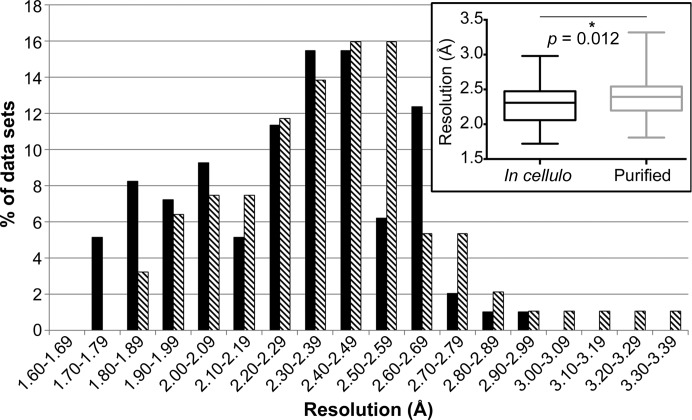
Distribution of diffraction resolution for *in cellulo* and purified polyhedra. Data from two independent crystal preparations analysed in two independent beamtime runs were pooled and represented as histograms (black, *in cellulo*; striped, purified) with a column graph as an inset. In the inset, the same data are analysed in a box-and-whisker plot: boxes represent the 25th and 75th percentiles, central lines represent the average value and whiskers indicate the minimum and maximum values. The *p* value indicates a significant difference between the two types of samples.

To compare *in cellulo* and purified crystals of the CPV1 polyhedrin, we compared these two types of crystals in parallel diffraction experiments. The quality of the data obtained from these experiments was then analysed using the *DISTL* software (Zhang *et al.*, 2006 ▸) to obtain a measure of the resolution limit of the diffraction for each crystal. A reproducible improvement in the average maximum resolution for the *in cellulo* crystals was observed for two biological replicates analysed in independent data-collection experiments. A first batch of crystals presented a clear difference between *in cellulo* and purified crystals, with average maximum resolutions of 1.95 Å (*n* = 20) and 2.14 Å (*n* = 23), respectively. While a repeat experiment carried out using a different batch of crystals showed generally lower resolution limits, the same trend was observed, with average maximum resolution limits of 2.37 Å for *in cellulo* (*n* = 74) and 2.47 Å for purified (*n* = 71) crystals. Overall, the improvement of the resolution for *in cellulo* crystals was small but statistically significant when compared with purified crystals analysed in optimum conditions (2.28 ± 0.29 Å *versus* 2.39 ± 0.29 Å, a significant difference with *p* = 0.012; Fig. 5 ▸).

The best data sets for *in cellulo* (*n* = 9) and purified (*n* = 7) crystals were obtained by scaling the most isomorphous crystals using a total of nine and seven crystals, respectively. Based on a cutoff criterion of signal to noise decreasing to below 2.0 in the last resolution shell, the maximum resolution for each data set was 1.55 Å for the *in cellulo* data set and 1.90 Å for the purified crystal data set. To provide another element of comparison, a second *in cellulo* data set was generated by using only the seven best *in cellulo* crystals and truncating the resolution to match that of the purified crystal data set (Table 1 ▸). Taken together, the statistics listed in Table 1 ▸ clearly indicate higher quality of either of the *in cellulo* crystal data sets compared with the purified crystal data set. For example, CC_1/2_ in the last shell is 91.3% *versus* 60.6% and the overall *R*_p.i.m._ is 7.5 and 17.3% for the two data sets processed at 1.90 Å resolution and collected from *in cellulo* and purified crystals, respectively. The resolution difference of 0.35 Å between the ‘best’ data sets is more significant than the shift observed in the average distribution of resolution limits of the data collected from *in cellulo* and purified crystals (Fig. 5 ▸), but is probably more representative of a typical data collection.

**Figure 6:**
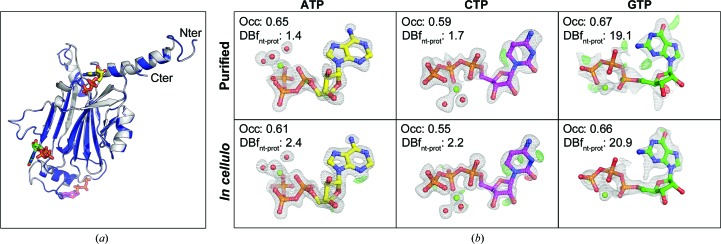
Comparison of the refined models derived from data collected from *in cellulo* or purified crystals. (*a*) Superposition of the two models refined against data from *in cellulo* crystals (coloured blue) and purified crystals (coloured grey). The protein models are represented in a ribbon representation with nucleotides shown as sticks coloured according to (*b*). The two models superpose almost perfectly, with an r.m.s.d. of 0.082 Å over all protein atoms. (*b*) Details of the nucleotides bound to the CPV1 polyhedrin. The nucleotides are shown as sticks with Mg atoms represented as green spheres and water molecules as red spheres. The 2*F*_o_ − *F*_c_ maps of the final refinement run are shown as a grey mesh with a threshold of 1.5σ; the corresponding *F*_o_ − *F*_c_ maps are shown as green and red meshes at thresholds of +3σ and −3σ, respectively. Occ., refined occupancies; DBf_nt-prot_, *B*-factor difference between nucleotide and protein atoms.

Structures were solved for the best data sets from each data-collection method using the previously reported CPV1 polyhedra structure as a template for rigid-body refinement (PDB entry 2oh5; Coulibaly *et al.*, 2007 ▸). Both structures were in the 100th percentile of best structures in *MolProbity* and refined to *R* and *R*_free_ values of 0.136 and 0.172, respectively, for the models derived from the *in cellulo* crystals and 0.149 and 0.201, respectively, for the purified crystals (Table 1 ▸). As reported in the first structure of the CPV1 polyhedrin, the ATP and CTP molecules have a very well defined electron density in the Fourier maps. The GTP molecule appears to be less well ordered, with a partially missing electron-density map around the ribose ring and higher average temperature factors. However, these features are comparable or better than the corresponding features in the deposited structures of CPV1 polyhedrin. The electron density corresponding to the three ligands shows slightly more detail in the *in cellulo*-derived density map (Fig. 6 ▸). The refined occupancies are similar in both structures, suggesting that in this case the better map can be attributed to the higher resolution of the data rather than better ligand retention of the ligands in cells.

## 6. Experimental phasing   

**Figure 7:**
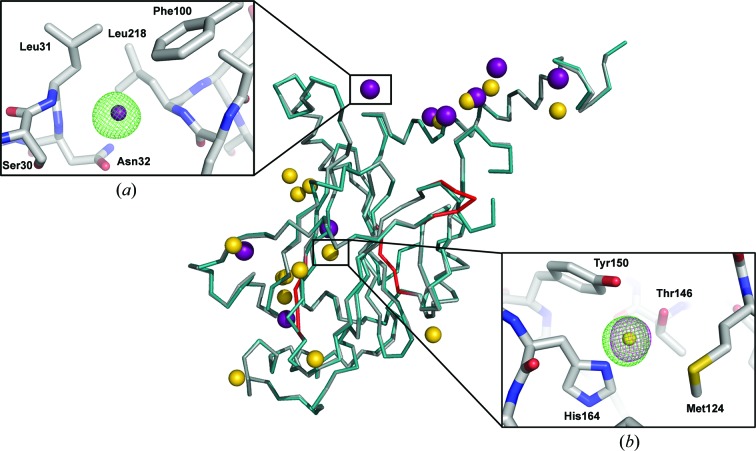
Experimental phasing of *in cellulo* data. Superposition of the model autobuilt by *Buccaneer* (coloured teal) with the final refined model (coloured grey). The regions of the autobuilt model that are missing or incorrectly built are highlighted in red on the final model. The iodine and gold sites identified and refined by *autoSHARP* are represented as purple and gold spheres, respectively. (*a*) Close-up of a selected iodine site, with the bound iodine represented as a purple sphere. The corresponding isomorphous map contoured at 5σ is shown as a green mesh. The anomalous map did not show peaks above 5σ. The neighbouring residues of the protein are shown as sticks and labelled. (*b*) Close-up of a selected gold site, with the bound gold ion shown as a yellow sphere. The corresponding anomalous and isomorphous maps contoured at 5σ are shown as pink and green meshes, respectively. The neighbouring residues are shown as sticks and labelled.

To assess the compatibility of the proposed pipeline with experimental phasing, sorted crystal-containing Sf9 cells were incubated with solutions of KAu(CN)_2_, KAuCl_4_, KAuBr_4_ or KI/I_2_, which were chosen on the basis of the heavy atoms reported to be incorporated into CPV1 polyhedra crystals (Coulibaly *et al.*, 2007 ▸). Data were collected above the energy of the *L*_III_ absorption edge of gold for most soaks or at a wavelength of 1 Å for iodine soaks. For all derivatives, anomalous maps and maps of isomorphous differences with the native data were inspected for the presence of electron-density peaks corresponding to the heavy atoms. Iodine incorporation into polyhedra was confirmed by peaks of >5σ in the isomorphous difference map. One of the sites corresponded to a binding site occupied by a chloride ion in the native structure. For the three gold salts, incorporation was confirmed by peaks in both anomalous and isomorphous maps (Fig. 7 ▸). For phasing, heavy-metal sites were located for each individual derivative data set using *SHELXC* (Sheldrick, 2008 ▸) and the substructure was refined using *SHARP* (Von­rhein *et al.*, 2007 ▸). The resulting sites were used to solve the CPV1 polyhedrin structure by the multiple isomorphous replacement with anomalous signal (MIRAS) method at 2.7 Å resolution (Table 2 ▸). Autobuilding with *Buccaneer* (Cowtan, 2008 ▸) at a resolution of 2.7 Å produced a model that is 93% complete and has an r.m.s.d. of 0.27 Å with the final *in cellulo* polyhedrin structure when all C^α^ atoms were compared.

## 7. Discussion   

Three recent studies have used *in cellulo* diffraction to determine the structures of intracellular crystals within their cellular context, namely CPV18 polyhedra and Xpa protein crystals using synchrotron X-ray radiation (Axford *et al.*, 2014 ▸; Tsutsui *et al.*, 2015 ▸) and Bt toxin crystals using an XFEL (Sawaya *et al.*, 2014 ▸). Our study extends these results by showing that analysis within the cellular environment rather than as purified crystals can be more efficient and can produce superior data.

Perhaps unexpectedly, a direct comparison between classical microcrystallography approaches and an *in cellulo* pipeline shows that the latter is not only more accessible to researchers with no previous expertise in microcrystallo­graphy, but is also more effective in terms of synchrotron time usage. For instance, a higher hit rate (96% *versus* 76%) means that more diffracting crystals can be collected from *in cellulo* polyhedrin crystals than from purified crystals for a set amount of beamtime. This relative simplicity results from the removal of a number of steps, including the identification of suitable conditions for cell lysis and crystal stabilization, crystal purification, cryoprotection and centring of individual microcrystals in the X-ray beam. Each of these steps represents an obstacle to the generalization of microcrystallographic approaches because of the knowhow required and the exploration of target-specific conditions. One step is added in this pipeline that consists of flow-sorting of crystal-containing cells. Because the *in cellulo* workflow involves handling cells rather than crystals, this step remains straightforward and can directly make use of standard flow-sorting methodologies that are often available as a platform facility. If a dedicated instrument is needed, this step is compatible with the new generation of benchtop cell sorters. These instruments are fully automated and, while still requiring instrument-specific training, remove the need for advanced skills in cell sorting. Although sorting criteria need to be refined for each sample, intracellular crystals typically lead to dramatic changes in the host-cell shape and protein distribution (Schönherr *et al.*, 2015 ▸), which creates well segregated populations compared with control cells.

The sorting methodology described here may also be useful in early stages for the high-throughput identification of constructs or targets that form *in vivo* crystals. Following sorting, populations enriched in cells that potentially generate crystals can be recovered for confirmation by imaging, SONICC (Kissick *et al.*, 2011 ▸) or X-ray diffraction experiments.

Importantly, the data collected *in cellulo* are of higher quality than the diffraction data obtained from purified crystals. A study by Axford and coworkers also found that the quality of the data was slightly better for crystals of CPV18 polyhedrin analysed *in cellulo* than for purified crystals as assessed by a reduced mosaic spread (Axford *et al.*, 2014 ▸). The quantitative analysis of CPV1 polyhedra presented here shows a very small difference in mosaic spread (0.17° *versus* 0.15°), but in this case the difference is not statistically significant. By contrast, the average resolution of diffracting crystals is reproducibly superior for *in cellulo* crystals and the resolution limit of the best data set is improved by ∼0.35 Å compared with purified crystals. Thus, removing most processing steps such as cell lysis, centrifugation and buffer exchange appears to be beneficial for polyhedrin crystals. Given the outstanding robustness of these crystals, one can anticipate a similar if not a greater protective effect for more sensitive microcrystals.

A major obstacle in microcrystallography remains phase determination where no suitable model is available. Thus, the finding that MIR phasing is compatible with *in cellulo* diffraction experiments and results in rapid structure determination in the case of the CPV1 polyhedrin provides new options for the determination of structures. We find that efficient derivatization of *in cellulo* crystals is possible despite the cellular envelope. Diffusion of the gold and iodine compounds inside the cell was anticipated given the rapid uptake of trypan blue by sorted cells which results from the loss of cell viability in the experimental setup adopted in this study. By contrast, the relative efficiency of heavy-atom binding is unexpected given the extreme density of CPV1 polyhedra, which have a solvent content of only 19% (Coulibaly *et al.*, 2007 ▸), and the high concentration of noncrystalline material available for off-target binding. The binding of heavy atoms to crystals within their cellular environment suggests that a similar approach could be applied to biologically relevant ligands at least as large as trypan blue (876 Da).

Overall, this comparative study suggests that in favourable cases the *in cellulo* approach can replace the complete workflow of structure determination with a pipeline that is not only fast and simple but also yields data of higher quality and that are potentially more relevant biologically. Of course, the CPV1 polyhedrin, like most examples of *in vivo*-grown crystals mentioned before, evolved to form crystals in the natural course of the infectious cycle. However, comparative analysis of such proteins has so far failed to uncover common factors between these proteins that may underlie their propensity to crystallize *in vivo*, which suggests that this process is accessible to proteins with diverse sizes, folds and surface features. Whether *in cellulo* analysis will provide an alternative to classical microcrystallography beyond the study of proteins that form crystals in their functional or pathological context now depends on advances on promoting *in vivo* crystallization and improving our ability to detect intracellular microcrystals.

## 8. Materials and methods   

### 8.1. Production of polyhedra-containing cells and purified polyhedra   

The cloning of recombinant *B. mori* CPV1 polyhedra and the production of virus stock in Sf9 insect cells were carried out as described previously (Mori *et al.*, 1993 ▸).

Sf9 cells were grown in suspension in Lonza Insect-XPRESS medium and split three times a week to 10^6^ cells ml^−1^ in a total of 200 ml. 24 h after splitting, the cells were infected with 1 ml P4 CPV1 polyhedrin stock. Sf9 cells were harvested 3 d post-infection and were kept on ice during the following steps unless specified otherwise.

### 8.2. Polyhedra purification   

After harvesting, 50 ml of infected Sf9 cells at about 8 × 10^6^ cells ml^−1^ were pelleted at 450*g* for 10 min. The supernatant was discarded and the pellet was resuspended in 40 ml phosphate-buffered saline (PBS) pH 7.4. The resuspended pellet was sonicated for 30 s at 10 mA using an MSE Soniprep 150 equipped with a 19 mm probe and centrifuged at 450*g* for 10 min. After centrifugation, two layers were observed: an upper pale brown layer consisting of debris and a lower white pellet consisting of polyhedra. The upper layer was removed by careful pipetting and the supernatant was discarded. The lower pellet was resuspended in PBS and subjected to further rounds of pelleting and resuspension until the polyhedra appeared clean when checked by light microscopy.

### 8.3. Cell sorting   

Propidium iodide was added to the cell suspension at a final concentration of 1 µg ml^−1^ just prior to flow-cytometric evaluation. Cells were sorted by flow cytometry using a BD Influx cell sorter (BD Biosciences) with a 100 µm nozzle (138 kPa with a frequency of 39 kHz). Crystal-containing cells were gated according to their differential high side scattering. Cells infected by a nonrelevant recombinant baculovirus were used as a control and did not have high side scattering.

The sorted cell populations were inspected for the presence of crystals in the cytoplasm using an IX71 inverted microscope (Olympus).

### 8.4. Data collection   

Data were collected at 100 K on the micro-crystallography beamline (MX2) at the Australian Synchrotron with a collimated X-ray beam of 10 × 10 µm. Using this setting, the flux was approximately 3.6 × 10^11^ photons s^−1^ at 13 keV. Data were collected at 12.4 keV unless specified otherwise, without attenuation. For both purified and *in cellulo* crystals, diffraction patterns were recorded with an exposure time of 10 s and an oscillation of 1° per image. Crystals were manually centred; for each putative crystal a test image was recorded followed by 10–15 further images if the test image showed diffraction to higher than 2.5 Å.

### 8.5. Diffraction-quality assessment   

Analysis was performed on data collected over two synchrotron visits, each of them using cells from different batches and prepared according to the pipeline, to assess (i) the proportion of usable data recorded after discarding the images showing no diffraction (owing to bad centring/missed crystal or intrinsically poor quality of the crystal) and (ii) the maximum resolution of each usable data set. Images were visually inspected and those that showed no diffraction were discarded. The *DISTL* software (Zhang *et al.*, 2006 ▸) was used to assess the diffraction quality and the resolution of the remaining data. *DISTL* was run on each test image (*i.e* the first image of the crystal). The pixel-array parameter was set to seven pixels and five pixels for the first and second run of data collection, respectively, because of the small size of the polyhedrin diffraction spots. The maximum resolution was limited to 1.8 Å. During run 1, data for crystal-containing cells were collected with or without trypan blue and ethylene glycol for comparison. Data were then collected without these two additives. Given that no effect of trypan blue and ethylene glycol was observed, the final data sets included data from the best crystals irrespective of whether trypan blue or ethylene glycol were present. Statistics were compiled and analysed with *GraphPad Prism* using t-tests (*GraphPad Prism* v.6, GraphPad Software, San Diego, California, USA; http://www.graphpad.com).

### 8.6. Data processing/structure determination   

Data collected from purified and *in cellulo* crystals were processed using the *HKL*-2000 package (Minor *et al.*, 2006 ▸). Subsequent data manipulation and model analyses were performed with the *CCP*4 suite (Winn *et al.*, 2011 ▸). The structure of CPV1 polyhedrin (PDB entry 2oh5; Coulibaly *et al.*, 2007 ▸) trimmed of water molecules and ligands was used as a model for rigid-body refinement using *BUSTER* 2.10 (Blanc *et al.*, 2004 ▸). Models were built in *Coot* (Emsley *et al.*, 2010 ▸) and were refined with *BUSTER* 2.10 (Blanc *et al.*, 2004 ▸). Data-processing and structure-refinement statistics are summarized in Table 1 ▸. The *R*_p.i.m._ values were generally high, presumably because data from a large number of crystals were merged to produce each data set presented here. The *R*_merge_ and *R*_p.i.m._ values are comparable to previous studies (Coulibaly *et al.*, 2007 ▸, 2009 ▸). A certain degree of non-isomorphism was observed and only crystals with good isomorphism with the highest resolution crystals were used. The final models and the corresponding structure factors were deposited in the Protein Data Bank as entries 5exy for *in cellulo* crystals and 5exz for purified crystals.

### 8.7. Cell derivatization with gold   

Saturated solutions of KAu(CN)_2_, KAuCl_4_, KAuBr_4_ and Au_2_Cl_6_ in PBS were prepared. After clarification by centrifugation, 20 µl of each solution was mixed with 55 000 sorted cells in 20 µl PBS. The half-saturated samples were kept at 4°C for 3 d. Data were collected at 13.9 keV, *i.e.* just above the *L*_II_ absorption edge of gold, as described previously, except that trypan blue was omitted since the cells were readily coloured by the gold solution. Although crystals incubated in Au_2_Cl_6_ did not diffract, three complete data sets were obtained after processing and scaling of the data collected from KAu(CN)_2_, KAuCl_4_ and KAuBr_4_ soaks at resolutions of 1.95, 2.50 and 2.70 Å, respectively.

### 8.8. Cell derivatization with iodine   

A stock solution was prepared by dissolving 1 g KI in 4 ml water and then adding 0.54 g I_2_. Derivatization was carried out using 30 000 sorted cells in 95 µl PBS, which were incubated with 5 µl KI/I_2_ stock solution for 3 d at 4°C. Data were collected at lower energy (8.5 keV) as previously described, except that trypan blue was omitted as the cells were readily coloured by the iodine solution. A 2.20 Å resolution complete data set was obtained after processing and scaling of the data.

### 8.9. Experimental phasing   

Isomorphous difference maps were calculated in *PHENIX* (Adams *et al.*, 2010 ▸) using the refined polyhedra model for phasing. Anomalous maps were created using the *CCP*4 suite (Winn *et al.*, 2011 ▸): anomalous difference data for each gold derivative were copied to the refinement output mtz file containing calculated structure factors, and the resulting mtz file was used to generate the map using *FFT* (Read & Schierbeek, 1988 ▸). Sites were identified independently using *SHELXC* (Sheldrick, 2008 ▸) and refined with *SHARP* (Von­rhein *et al.*, 2007 ▸) at a resolution of 2.7 Å. The solvent-flattened map was used for autobuilding with *Buccaneer* (Cowtan, 2008 ▸) using experimental phases at a resolution of 2.7 Å. Phasing statistics are summarized in Table 2 ▸.

*Note added in press.* While this article was in press a study by Jakobi and colleagues reported another example of *in cellulo* serial crystallography relevant to the research presented here (Jakobi *et al.*, 2016 ▸).

## Supplementary Material

PDB reference: CPV1 polyhedrin, *in cellulo* structure, 5exy

PDB reference: structure from purified crystals, 5exz

## Figures and Tables

**(a):** Data collection.

	Purified	*In cellulo*	*In cellulo*, 1.9 Å resolution, seven crystals
Wavelength (Å)	1.0000	1.0000	1.0000
No. of crystals	7	9	7
Resolution range (Å)	30–1.90 (1.97–1.90)	30–1.55 (1.61–1.55)	30–1.90 (1.97–1.90)
Space group	*I*23	*I*23	*I*23
Unit-cell parameters (Å)	*a* = *b* = *c* = 103.2 (σ = 0.1)	*a* = *b* = *c* = 103.2 (σ = 0.1)	*a* = *b* = *c* = 103.2 (σ = 0.1)
Measured reflections	45800	155448	78973
Average mosaicity (°)	0.17 (σ = 0.04)	0.16 (σ = 0.05)	0.16 (σ = 0.05)
Multiplicity†	3.3 (3.4)	5.9 (4.8)	5.5 (5.5)
〈*I*〉/〈σ(*I*)〉†	4.5 (1.9)	7.6 (1.9)	10.6 (6.0)
Completeness† (%)	94.5 (93.4)	99.6 (99.4)	99.5 (99.6)
*R*_p.i.m._ †	0.173 (0.398)	0.111 (0.466)	0.075 (0.150)
CC_1/2_ †	(0.606)	(0.519)	(0.913)

**(b):** Refinement.

	Purified	*In cellulo*	
	No hydrogen	No hydrogen	H atoms
Resolution range† (Å)	28–1.90 (2.05–1.90)	24–1.55 (1.61–1.55)	24–1.55 (1.61–1.55)
No. of reflections†	13732 (2753)	26475 (2903)	26475 (2903)
*R*/*R*_free_ †	0.147/0.204 (0.174/0.239)	0.144/0.184 (0.190/0.230)	0.128/0.167 (0.181/0.226)
No. of atoms			
Protein	2003	2012	3925
Ligands	99	96	138
Solvent	173	189	203
R.m.s. deviation, bonds (Å)	0.010	0.010	0.010
R.m.s. deviation, angles (°)	1.04	1.04	1.08
Average *B* factor (Å^2^)			
Protein	6.9	7.3	7.5
Ligands	14.4	15.7	16.7
Waters	12.0	14.8	16.0
Ramachandran plot			
Favoured (%)	98.4	97.2	97.6
Allowed (%)	1.6	2.8	2.4
Outliers (%)	0.0	0.0	0.0

† Values in parentheses are for the outer resolution shell.

**Table 2:** Data-processing and phasing statistics for the *in cellulo* derivatives

	Native	KAu(CN)_2_	KAuBr_4_	KAuCl_4_	KI/I_2_
Data collection					
Wavelength (Å)	1.0000	0.8920	0.8920	0.8920	0.9537
No. of crystals	9	8	10	12	18
Resolution range† (Å)	30–1.55 (1.61–1.55)	30–1.95 (2.02–1.95)	25–2.70 (2.80–2.70)	30–2.50 (2.59–2.50)	30–2.20 (2.28–2.20)
Space group	*I*23	*I*23	*I*23	*I*23	*I*23
Unit-cell parameters (Å)	*a* = *b* = *c* = 103.2 (σ = 0.1)	*a* = *b* = *c* = 103.2 (σ = 0.2)	*a* = *b* = *c* = 103.3 (σ = 0.1)	*a* = *b* = *c* = 103.4 (σ = 0.1)	*a* = *b* = *c* = 103.4 (σ = 0.2)
Measured reflections	155448	53966	30027	44688	44179
Average mosaicity (°)	0.16 (σ = 0.05)	0.14 (σ = 0.05)	0.15 (σ = 0.03)	0.12 (σ = 0.03)	0.17 (σ = 0.05)
Multiplicity†	5.9 (4.8)	4.1 (2.2)	5.9 (5.4)	7.0 (2.2)	4.8 (3.2)
〈*I*〉/〈σ(*I*)〉†	7.6 (1.9)	5.9 (2.0)	6.5 (3.5)	6.9 (2.3)	5.7 (2.1)
Completeness† (%)	99.6 (99.4)	97.0 (82.8)	99.4 (99.0)	98.4 (88.5)	97.9 (94.6)
*R*_p.i.m._ †	0.111 (0.466)	0.142 (0.361)	0.139 (0.268)	0.119 (0.367)	0.143 (0.372)
CC_1/2_	(0.519)	(0.676)	(0.785)	(0.678)	(0.663)
Phasing					
Resolution range (Å)	19–2.70				
No. of sites		8	10	9	9
Phasing power, isomorphous (acentric/centric)					
Overall		1.13/0.90	2.10/1.42	1.91/1.24	0.76/0.55
18.84–9.86 Å shell		2.19/0.86	4.15/1.83	3.41/1.21	0.82/0.52
2.78–2.70 Å shell		0.63/0.52	1.29/1.09	1.54/1.31	0.59/0.41
Phasing power, anomalous					
Overall		0.27	0.48	0.72	0.10
18.84–9.86 Å shell		1.03	1.94	2.28	0.23
2.78–2.70 Å shell		0.15	0.36	0.53	0.07
FOM (acentric/centric)					
Overall	0.66/0.72				
18.84–9.86 Å shell	0.92/0.84				
2.78–2.70 Å shell	0.52/0.66				
Autobuilding					
Resolution range (Å)	72–2.70				
Residues built/sequenced (%)	96.8/92.6				
*R*/*R*_free_	0.240/0.348				

† Values in parentheses are for the outer resolution shell.
